# A hybrid ensembling and autoencoder scheme for improving sensing reliability in cognitive radio networks

**DOI:** 10.1371/journal.pone.0338915

**Published:** 2025-12-30

**Authors:** Noor Gul, Jehad Ali, Sana Ul Haq, Junsu Kim, Su Min Kim

**Affiliations:** 1 Department of Electronics, University of Peshawar, Peshawar, Khyber Pakhtunkhwa, Pakistan; 2 Department of AI Convergence Network, Ajou University, Suwon, Republic of Korea; 3 Department of Electronics Engineering, Tech University of Korea, Siheung, Gyeonggi, Republic of Korea; Beijing Institute of Technology, CHINA

## Abstract

This paper proposes a hybrid ensemble classifier with denoising autoencoder (ECDAE) framework to address reliability and robustness challenges in cooperative spectrum sensing (CSS) for cognitive radio networks (CRNs). The proposed framework first employs an ensemble classifier (EC) to dynamically reconfigure the sensing time, optimizing performance while minimizing cost. The EC accurately estimates the sensing samples based on target detection probabilities, false alarm rates, and channel conditions. Subsequently, a denoising autoencoder (DAE) eliminates soft-combined energies from false-sensing users (FSUs) before soft fusion. The results show that EC surpasses other methods, including random forest (RFC), neural networks (NN), decision trees (DT), k-nearest neighbors (KNN), Gaussian naive Bayes (GNB), RUSBoost and XGBoost, achieving an F1 score of 99.23%, an accuracy of 99.78%, and a Matthews correlation coefficient (MCC) of 99.6%. Furthermore, optimized sensing time through EC is combined with DAE reconstruction delivers superior sensing performance at the fusion center (FC) producing low error probabilities compared to traditional schemes such as identical gain combination (IGS), highest gain combination (HGS), particle swarm optimization (PSO), differential evolution-based machine learning (DE-ML) and convolutional neural networks (CNN). On average, the ECDAE framework achieves a 99.4% and 98.1% reduction in error probability compared to traditional schemes (IGS, HGS) and a 92.7% to 97.2% reduction compared to advanced methods (PSO, DE-ML, CNN), across all tested SNR conditions and false-sensing attack scenarios. The framework maintains robustness across four distinct false-sensing scenarios: (1) no false sensing (NFS) reporting an low-energy signals, (2) yes false sensing (YFS) reporting consistently an always high-energy signals, (3) opposite false sensing (OFS) reporting an always invert decisions to the true energy states, and (4) yes/no false sensing (YNFS) where FSU randomly alternates between YFS and NFS - ensuring minimal error probabilities in global decisions.

## 1 Introduction

In CR systems, spectrum sensing is a critical phase that relies on user collaboration for optimal performance. However, multipath fading and shadowing effects can degrade the accuracy of sensing. There are two primary cooperative schemes for reliable primary user (PU) channel detection: centralized and distributed. This study focuses on the centralized approach, where a fusion center (FC) collects sensing reports from multiple users to make a final decision [1].

Given the centrality of cooperative sensing in CR, its effectiveness is critically important. This is especially true in the growing demand for wireless spectrum, which is driven by 5G and mobile devices and requires efficient spectrum management through cognitive radio networks (CRNs) [2]. To address these demands, researchers have developed advanced optimization techniques, such as a novel hybrid algorithm that integrates the firefly algorithm, the genetic algorithm (GA), and ant colony optimization (ACO) for TV white space networks [3]. It outperforms traditional hybrid methods in spectrum allocation, enhancing throughput and objective function values. Beyond optimization, efficient spectrum management also relies on robust sensing methods. This is exemplified by LASSO-based compressive sensing, an effective solution for wideband spectrum sensing in 5G networks [4]. This method demonstrates strong signal recovery capabilities that are critical for contemporary spectrum management.

However, the benefits of cooperative sensing are undermined by security challenges. Cooperative sensing is beneficial in CR networks, but the presence of false sensing users (FSUs) raises security concerns for the collaborative network. FSUs may deliberately provide incorrect sensing information to the FC to disrupt global decisions and potentially gain access to valuable spectrum resources [5]. These threats are multifaceted: Research studies have explored different types of false and misleading users that could jeopardize the FC’s decision. This includes individuals with false intent, such as malicious users (MUs), PU emulation attackers (PUEAs), Byzantine users, and jammers [6–9]. In contrast, collusion attacks comprise multiple attackers that combine to launch more powerful attacks against the FC [10,11].

### 1.1 Related work

In recent years, spectrum sensing techniques have transitioned from traditional methods such as energy detection to more advanced approaches based on machine learning (ML), deep learning (DL), reinforcement learning (RL), and artificial intelligence (AI). These advanced techniques significantly improve detection accuracy and robustness, especially in dynamic and noisy conditions, where traditional methods often struggle [12]. They improve CRN performance by automatically detecting environmental signal patterns and making accurate spectrum availability decisions. In [13] an ensemble learning framework has been introduced for cooperative spectrum sensing (CSS), highlighting significant improvements in detection and false alarm probabilities compared to conventional methods. The K nearest neighbor (KNN) algorithm in [14] has examined the accuracy, sensitivity, and confusion matrix to evaluate the classification of the PU channel. Similarly, multiple ML techniques, including KNN, are proposed in [15] to detect PU emulations in a mobile CRN environment. This method employs a prior learning process to create datasets using the SNR and received power entropy. In particular, it operates without the need for prior knowledge of modulation types or RF characteristics.

Building on advances in ML, recent work demonstrates how DL and convolutional neural networks (CNNs) can optimize spectrum sensing [16]. The integration of CNNs and recurrent neural networks (RNNs) in sensing has demonstrated superior performance over traditional methods, capturing spatial and temporal patterns in CR systems [17,18]. In [19], a novel DL-based sensing detector utilizing CNN and long-short-term memory (LSTM) is proposed to exploit energy correlation features and PU patterns without making assumptions from the signal-noise model. DL is also used to improve the classification of sensing by incorporating prior knowledge of PU with LSTM layers in [20]. The authors in [21] develop a multi-agent reinforcement learning scheme using distributed algorithms to help cognitive unmanned aerial vehicles (UAVs) decisions by linking sensing and access approaches. In this framework, the sensing phase is performed cooperatively, with binary decisions being exchanged between agents to inform the joint access strategy. Similarly, in [22], an RL-based CSS scheme improves the sensing performance in dynamic CR networks. In this approach, each SU acts as an agent that learns the behavior of channels and neighbors to improve detection efficiency, decrease scanning overhead, and reduce access delay.

Virtual CSS (VCSS) proposed in [23] tried to overcome power limitations in UAV networks by utilizing sequential decision fusion and ML techniques. The network in [24] is trained using the multilayer perceptron model to reduce the prediction error and mitigate the impact of collision factors. Furthermore, CNN-based method in [25] processes input from multiple secondary users (SUs). This approach specifically addresses how the combination of diverse sensing affects the detection performance. Reference [7] presents a GA method that computes optimal weighting coefficients for soft combination. These weights are then applied in soft decision fusion (SDF) for final decisions. Furthermore, [26] presents a hopping sequence module to increase the probability of detection when PUs and SUs are mobile. Finally, [27] and [28] use CSS with spatial variability in the classification of SU using SVM to reduce uncertainty in the global decision. Table 1 provides a summary of related work.

**Table 1:** Literature summary of advanced spectrum sensing techniques.

Research Topic	Approach	Limitations	References
Transition to AI-based methods	ML/DL/RL for accuracy in noisy environments	- Lacks quantitative comparisons under FSU attacks	[12]
Cooperative spectrum sensing	Ensemble learning framework	- Static sensing parameters - No FSU attack evaluation - Lacks denoising	[13]
PU channel classification	KNN with accuracy/sensitivity metrics	- Single-model vulnerability - No adversarial analysis - Fixed sensing framework	[14]
PUEA detection in mobile CRNs	ML using SNR/entropy features	- Narrow threat model (only PUEAs) - No sensing optimization	[15]
DL-based efficiency improvement	CNNs/RNNs for spatiotemporal patterns	- Signal-specific (OFDM-only) - High computational load - Untested under attacks	[16–18]
DL sensing detector	CNN-LSTM for energy features	- No FSU resilience - Fixed sensing window - Heavy computation	[19]
Spectrum prediction	RNN for multi-time-slot forecasting	- Not real-time sensing - Ignores FSUs	[20]
MARL for UAV CRNs	Joint sensing-access optimization	- UAV-specific - No FSU mitigation - MARL overhead	[21]
RL-based CSS	SU learning of channel behavior	- Fixed sensing parameters - RL training overhead - No FSU analysis - Assumes honest reporting	[22]

Multi-user CSS has emerged as a promising approach. However, existing cooperative methods face several challenges. Traditional methods often use fixed sensing times, which can lead to inefficiencies in spectrum utilization and reduced throughput. Similarly, the presence of FSUs in CSS degrades both the sensing performance and the error probabilities. To address these limitations, we propose a hybrid scheme that combines an ensemble classifier (EC) and a denoising autoencoder (DAE). The EC dynamically adjusts the sensing time, improving the efficiency and network throughput, while the DAE cleans the sensing data from FSU disturbances and noisy channel conditions, improving the sensing reliability.

In the cooperative model, increasing the number of sensing samples (more sensing time) leads to higher sensing accuracy. However, this also results in increased energy consumption and reduced channel throughput. In contrast, optimal choice of the sensing sample for the individual and the user in cooperation leads to benefits such as improved throughput, low sensing cost, and guaranteed sensing with minimum error. A similar problem of multichannel detection without the use of AI tools is investigated in [29,30]. The authors of [31] analyzed the performance of CSS in the presence of FSUs using a particle swarm optimization (PSO) scheme. In contrast, the work in [32] focused on a hybrid differential evolution-based ML (DE-ML) scheme to improve the FC decision with fixed sensing samples, but lacks denoising effects.

**Fig 1:**
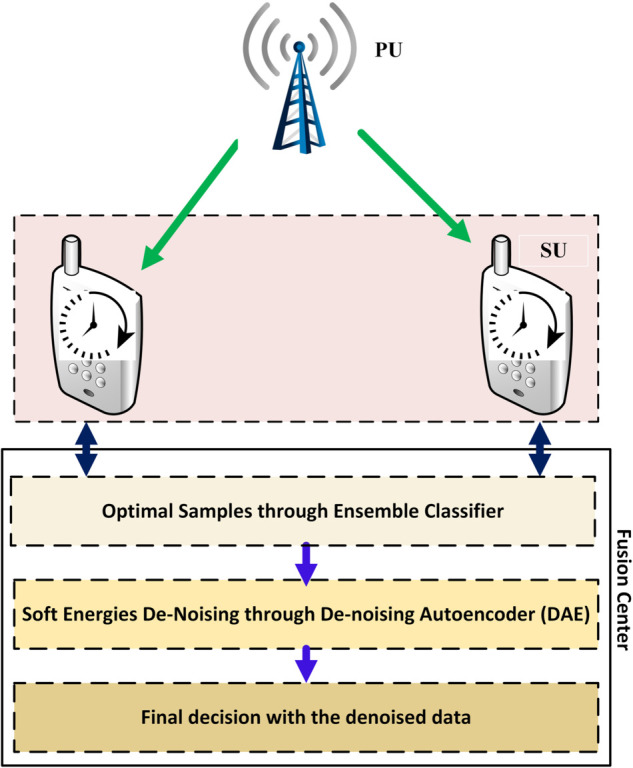
CSS with reconfigurable sensing time.

While existing approaches demonstrate progress, they possess critical limitations that are systematically summarized in Table 1. Our ECDAE framework bridges these gaps through : (1) dynamic sensing-time optimization through ensemble learning that adapts to real-time channel conditions, (2) integrated DAEs that purify sensing reports from both FSU distortions and channel noise with low latency, and (3) comprehensive validation across various FSU attack scenarios, demonstrating 82-100% error reduction compared to state-of-the-art methods. This paper is an extension of our previous work [33]. In this paper, a hybrid ensemble classifier-based DAE (ECDAE) scheme is suggested that integrates the ensemble classifier with the autoencoder as in Fig 1. The main contributions of this paper are summarized as follows:

- **Novel Hybrid Architecture:** We propose the first framework, to our knowledge, that integrates an ensemble classifier (EC) with a DAE for cooperative spectrum sensing. This ECDAE architecture uniquely addresses both sensing-time optimization and data purification in a single cohesive system.
- **Dynamic Sensing-Time Optimization:** We introduce an EC-based mechanism that dynamically reconfigures the sensing time for each user. This optimization is based on real-time signal-to-noise ratio (SNR) conditions and target detection (*P*_*d*_) and false alarm (*P*_*f*_) probabilities, significantly enhancing spectrum utilization efficiency and minimizing sensing overhead.
- **Robustness Against sensing Attacks:** Unlike prior work that often assume benign conditions, our framework is rigorously evaluated against a comprehensive suite of false-sensing user (FSU) attacks, including always no (NFS), always yes (YFS), always opposite (OFS) and random yes/no (YNFS) reporting strategies, ensuring practicality for adversarial real-world environments.
- **Data Denoising:** We employ a dedicated DAE to cleanse the aggregated sensing data at the FC. This step effectively mitigates distortions caused by both additive white Gaussian noise (AWGN) and malicious data injections from FSUs, providing a reliable input for the final decision fusion.
- **Performance Validation:** Through extensive simulations, we demonstrate that our proposed ECDAE framework consistently outperforms a wide range of state-of-the-art techniques, including traditional combination schemes (IGS, HGS), optimization algorithms (PSO, DE-ML), and deep learning models (CNN), across different tested attack scenarios and SNR conditions.

## 2 System model and preliminaries

The system model has *m* reporting users, including regular and fraudulent. The users sense the channel and report the presence and absence information to the FC. This work holds a single PU channel and employs an energy detector for spectrum sensing. The *H*_0_ and *H*_1_ hypothesis test of the *j*^*th*^ user as in Table 2 is as follows:

$$\left\{\begin{array}{c} H_{0},\;\;x_{j}(l)=v_{j}(l)\\ H_{1},\;\;x_{j}(l)=g_{j}c(l)+v_{j}(l) \end{array}\right\},j\in \left\{1,2,...,m\right\},l\in \left\{1,2,...,k\right\}.$$ (1)

*g*_*j*_ denotes the channel gain for the *j*^*th*^ user. The channel gain *g*_*j*_ is not constant but varies due to fading and shadowing effects. This variation is modeled using a Rayleigh distribution for fading and a log-normal distribution for shadowing, which are commonly used in wireless communication systems. *c*(*l*) is the PU signal in the *l*^*th*^ slot. $v_{j}(l)$ is Gaussian noise with a mean of zero and variance $\sigma_{v_{j}}^{2}$. The sensing energy of the users has a total of $k=2B\tau_{s}$ sensing samples. Here, *B* is the bandwidth with $\tau_{s}$ sensing period. The energy consumption for (1) with *k* sensing samples is derived as

$$E_{j}(i)=\left\{\begin{array}{c} \sum_{l=l_{i}}^{l_{i}+k-1}{\left|v_{j}(l)\right|}^{2},\;\;\;\;\;\;\;\;H_{0}\\ \sum_{l=l_{i}}^{l_{i}+k-1}{\left|g_{j}c(l)+v_{j}(l)\right|}^{2},\;H_{1} \end{array}\right\},$$ (2)

**Table 2:** List of mathematical notations.

Term	Description
*H*_0_, *H*_1_	Hypothesis tests: *H*_0_ (PU absent), *H*_1_ (PU present)
*m*	Total number of cooperative users
*g*_*j*_	Channel gain for the *j*^*th*^ user
*c*(*l*)	PU signal in the *l*^*th*^ slot
$v_{j}(l)$	Gaussian noise with mean zero and variance $\sigma_{v_{j}}^{2}$
*k*	Total sensing samples, $k=2B\tau_{s}$
*B*	Bandwidth of the communication system
$\tau_{s}$	Sensing period during which users collect sensing samples
*E*_*j*_	Sensing energy of the *j*^*th*^ user
$\left(\mu_{0},\;\sigma_{0}^{2}\right)$	Mean and variance of the energy distribution under *H*_0_ hypothesis
$\left(\mu_{1},\;\sigma_{1}^{2}\right)$	Mean and variance of the energy distribution under *H*_1_ hypothesis
$\eta_{j}$	SNR of the *j*^*th*^ user
*T*	Total time duration for sensing and data transmission
β	Threshold for the energy detector
*P*_*f*_	False alarm probability
*P*_*d*_	Detection probability under *H*_1_ hypothesis
Q(.)	Complementary Gaussian distribution function
$Q^{-1}\left(.\right)$	Inverse complementary Gaussian distribution function
$t_{1},t_{2},t_{n}$	Feature vectors
*T*	Training set matrix of size, $T\in {ℜ}^{n\times (m+1)}$
*X*,*Y*	Feature and label matrices
*r*	Classifier number
*h*_*p*_	Predicted value of the *p*^*th*^ classifier
*g*	denotes the nonlinear activation sigmoid function
$\alpha_{p}$	Weight assigned to the *p*^*th*^ classifier prediction
ψ	DAE optimal parameters
*C*_*HGS*_	Cumulative decision using highest gain combination
*C*_*ECDAE*_	Cumulative decision using ensemble classifier based DAE

The soft energy reports under satisfactory sensing samples converge to the Gaussian random variable as

$$E_{j}\sim \left\{\begin{array}{c} N\left(\mu_{0}=k,\sigma_{0}^{2}=2k\right),\;\;\;\;\;\;\;\;\;\;\;\;\;\;\;\;\;\;\;\;\;\;\;\;\;\;\;\;H_{0}\\ N\left(\mu_{1}=k(\eta_{j}+1),\sigma_{1}^{2}=2k(\eta_{j}+1)\right),\;\;\;H_{1} \end{array}\right\}.$$ (3)

**Fig 2:**
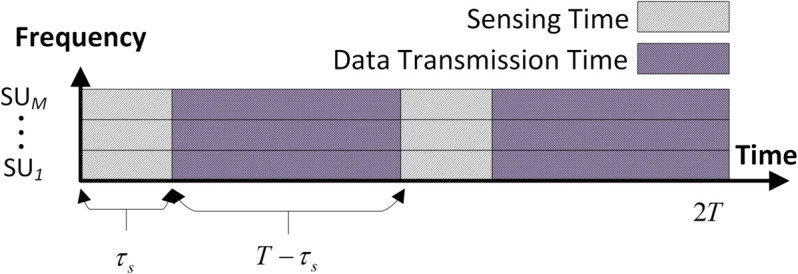
Frame structure.

Here, $\left(\mu_{0},\;\sigma_{0}^{2}\right)$ and $\left(\mu_{1},\;\sigma_{1}^{2}\right)$ are the mean and variance results of the energy distribution, while $\eta_{j}$ is *j*^*th*^ user SNRs. Fig 2 shows the duration of user sensing and data transmission time as $\tau_{s}$ and $T-\tau_{s}$ when the channel is free. The sensing time between the cooperative users is assumed to be synchronized.

In self-dependent sensing, each user makes local sensing decisions to infer PU activity. In cooperative sensing, sensing participants report their observations to a data collection center, such as a FC, which aggregates the data to form a global decision. The FC is assumed to provide feedback to the sensing users regarding its final decision. In addition, a dedicated control channel is assumed to facilitate communication between the SU and the FC.

**Fig 3:**
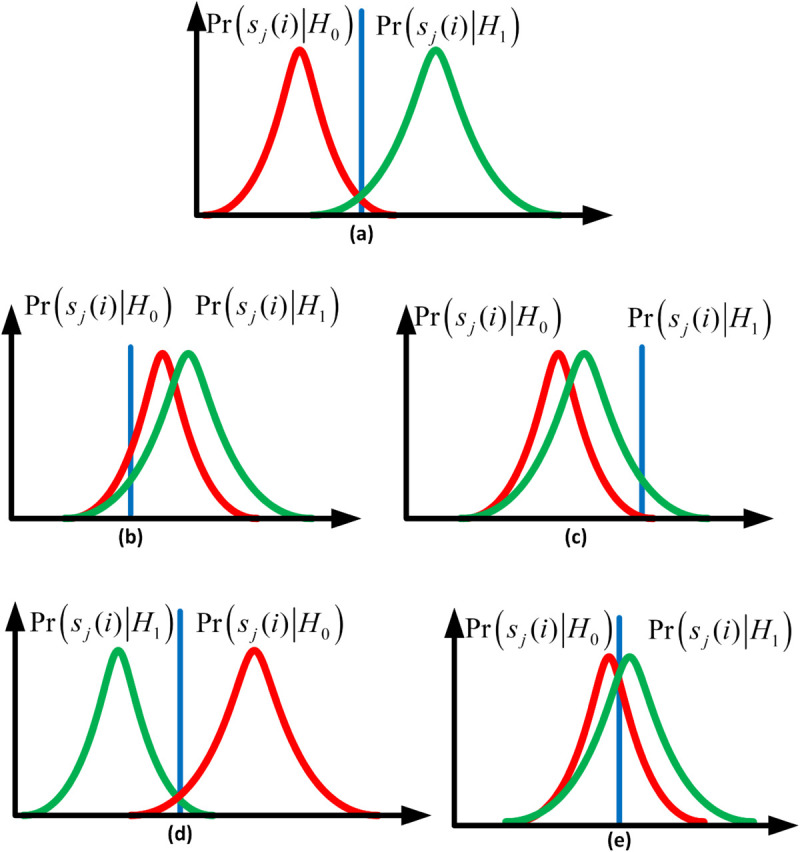
Probability distribution function: (a) honest sensing (b) YFS (c) NFS (d) OFS (e) YNFS.

The cooperative environment in this paper is also protected against false detection notices from YFS, NFS, OFS, and YNFS users. Fig 3 illustrates the distinct energy distributions for honest and FSUs. Honest SUs report higher energies under *H*_1_ and lower under *H*_0_. In contrast, FSUs exhibit malicious patterns: YFS consistently reports high energy, NFS consistently reports low energy, OFS inverts the true state, and YNFS randomly alternates. These behaviors degrade network performance: YFS reduces SU throughput by causing false alarms, while NFS, OFS, and YNFS cause harmful interference to the PU by increasing missed detections. The detailed energy statistics for reporting users are provided in S1 File

### 2.1 Attacker model and threat assumptions

To rigorously evaluate the robustness of the proposed ECDAE framework, we formalize the attacker model based on the FSU types introduced in Fig 3. This model defines the capabilities, knowledge, and goals of the adversaries.

- System Knowledge: FSUs are assumed to have perfect knowledge of the global hypothesis (*H*_0_ or *H*_1_) for each sensing interval. This represents a worst-case scenario where attackers can perfectly monitor the PU channel, allowing them to execute targeted attacks. This strong assumption ensures that our model is robust against highly informed adversaries.
- Report Manipulation: FSUs can arbitrarily manipulate their local soft energy reports *E*_*j*_ before transmitting them to the FC. They are not limited to simple binary decisions but can generate continuous-valued false energy statistics that conform to expected distributions (e.g., Gaussian with manipulated mean/variance), making their reports appear statistically believable.
  The primary objective of the FSUs is to disrupt the FC’s global decision. The specific goals vary by attack type:
- NFS Goal: Maximize the probability of missed detection (*P*_*m*_). By consistently reporting low energy, the attacker aims to deceive the FC to declare the channel free (*H*_0_) even when the PU is active (*H*_1_), causing harmful interference to the PU.
- YFS Goal: The attacker’s goal is to maximize the probability of false alarm (*P*_*f*_). They do this by consistently reporting high energy to deceive the Fusion Center (FC) into declaring a busy channel (*H*_1_) when it is actually free (*H*_0_). This illegally monopolizes the spectrum and reduces SU throughput.
- OFS Goal: Maximize the total probability of error (*P*_*e*_). By always reporting the opposite of the true state, the attacker seeks to make the FC’s decision uniformly unreliable, maximizing both *P*_*m*_ and *P*_*f*_ regardless of the actual PU activity.
- YNFS Goal: The attacker’s objectives are to create uncertainty and avoid detection. By randomly switching strategies, they become less predictable than a pure NFS or YFS attacker. This randomness helps them evade simple outlier detection schemes, while still degrading overall sensing performance.

This comprehensive attacker model establishes a strong foundation for evaluating the ECDAE framework in a stringent and realistic threat environment, ensuring that the demonstrated robustness is credible and meaningful.

To address the dual challenges of sensing efficiency and robustness against the FSU attacks described in this model, we propose a hybrid ECDAE framework that operates sequentially in two distinct phases. The first phase is called the Sensing-Time Configuration. In it, an EC dynamically finds the optimal sensing time *k*_*j*_ for each user *j*. This ensures that the user meets its target *P*_*d*_ and *P*_*f*_ requirements based on the current SNR conditions $\eta_{j}$. In the second phase, data denoising and fusion, users sense the channel using their assigned *k*_*j*_ and report soft energy statistics *E*_*j*_ to the FC. A DAE then processes the aggregated report vector $𝐬=[E_{1},E_{2},...,E_{m}]$. Its purpose is to mitigate distortions from both AWGN and FSUs. The output is a cleaned vector $\hat{𝐬}$, which is used for a reliable final decision fusion. The specific details of each phase and their interface are elaborated in the following section.

## 3 Optimal sensing time estimation and data denoising

**Fig 4:**
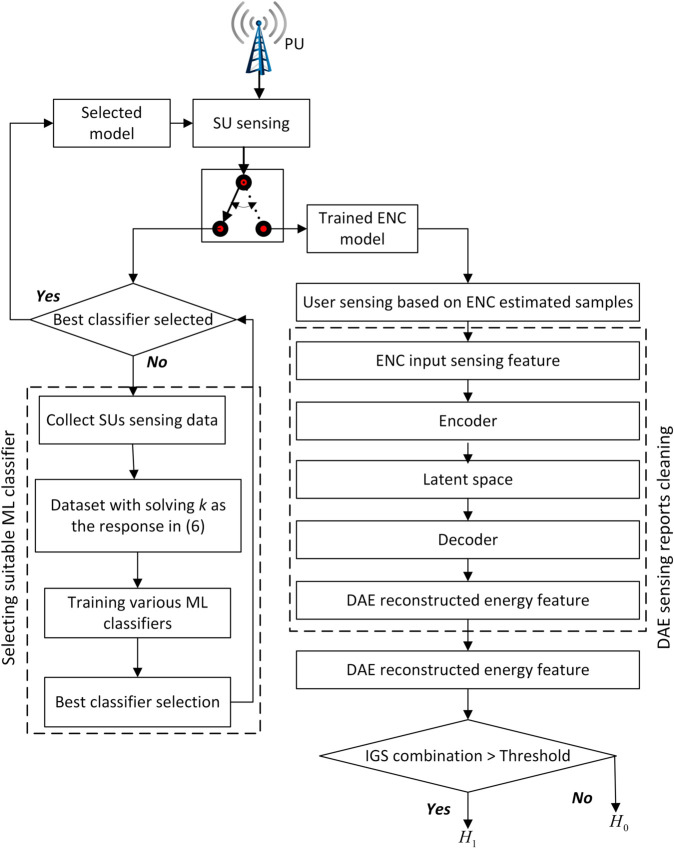
Flowchart diagram.

The proposed ECDAE framework, illustrated in Fig 4, operates as a sequential pipeline. The output of the first phase (EC) serves directly as the configured parameter for the second phase (sensing), whose output is then processed by the third phase (DAE). The interface between the theoretical model and the scheme, and between the phases, is formalized as follows:

*Input*: The framework takes as input the system parameters, the number of users *m*, their SNRs $\eta_{j}$, and the network target performance metrics (*P*_*d*_, *P*_*f*_).

*Phase 1: EC for Sensing-Time Configuration*: The pre-trained EC model uses the tuple $\left(\eta_{j},P_{f},P_{d}\right)$ for each user to predict the optimal number of sensing samples *k*_*j*_. This completes the first phase.

*Interface 1 (Phase 1 → Phase 2)*: The values *k*_*j*_ are communicated to the respective SUs. This is the critical hand-off between the two core algorithmic components.

*Phase 2: Sensing and Reporting*: Each SU *j* performs energy detection as defined in Eq (2), but now uses its optimized sample size *k*_*j*_ instead of a fixed value. It then calculates its energy statistic *E*_*j*_ as defined in Eq (3) and reports it to the FC.

*Interface 2 (Phase 2 → Phase 3)*: The FC aggregates these reports into a vector $𝐬=[E_{1},E_{2},...,E_{m}]$, which forms the input to the next phase.

*Phase 3: DAE for Denoising*: The pre-trained DAE takes the potentially corrupted vector **s** and reconstructs a cleaned version $\hat{𝐬}$, effectively removing the influence of AWGN and FSU attacks.

The cleaned vector $\hat{𝐬}$ is then used in a soft combination rule to make a global decision on the presence of PU. This sequential process ensures that the sensing is first optimized for efficiency before the reports are purified for robustness, addressing both core challenges independently but cohesively.

### 3.1 Dataset for reconfigurable sensing time

This section describes the construction of a dataset to estimate the optimal number of samples for the ML classifier. Sensing samples used in energy reports (3) are determined for a target false alarm, SNR, and detection probability requirements as

$$P_{f}=Q\left(\frac{\left(\beta -\mu_{0}\right)}{\left(\sqrt{\sigma_{0}^{2}}\right)}\right)=Q\left(\frac{\left(\beta -k\right)}{\left(\sqrt{2k}\right)}\right),$$ (4)

where β is the threshold for the energy detector, *P*_*f*_ is the false alarm probability, while $\mu_{0}$ and $\sigma_{0}^{2}$ are the mean and variance. The expression of the detection probability *P*_*d*_ in the possibility of the PU presence hypothesis *H*_1_ is as follows:

$$P_{d}=Q\left(\frac{\left(\beta -\mu_{1}\right)}{\left(\sqrt{\sigma_{1}^{2}}\right)}\right)=Q\left(\frac{\left(\beta -k\left(\eta_{j}+1\right)\right)}{\left(\sqrt{2k\left(\eta_{j}+1\right)}\right)}\right),$$ (5)

Eqs (4) and (5) are standard expressions for the false alarm probability (*P*_*f*_) and the detection probability (*P*_*d*_) in energy detection, as derived in [34]. Likewise, the expression of the detection probability is expressed as SNRs, the number of samples, and the false alarm probability as follows [35]:

$$P_{d}=Q\left(\frac{\left(\sqrt{2}Q^{-1}\left(P_{f}\right)-\sqrt{k}\eta_{j}\right)}{\left(\sqrt{2\left(\eta_{j}+1\right)}\right)}\right).$$ (6)

Eq (6) expresses the detection probability (*P*_*d*_) in terms of the false alarm probability (*P*_*f*_), SNR and sensing samples (*k*). This formulation is based on the complementary and inverse complementary Gaussian distribution functions, as described in [36]. Q(.) and $Q^{-1}\left(\;.\;\right)$ are the complementary and inverse complementary Gaussian distribution functions. Eq (6) needs a non-convex function solution to find optimal sensing samples for a given detection, false alarm, and SNR conditions, which is difficult to obtain in real time. The nonconvexity of (6) arises from the inverse complementary Gaussian distribution function ($Q^{-1}(\cdot )$), which does not have a closed form solution. Solving this equation analytically is computationally expensive and impractical for real-time applications. To address this challenge, we construct a dataset that maps the input parameters ($\eta_{j}$, *P*_*f*_, *P*_*d*_) to the optimal sensing samples (*k*). This dataset is generated by solving (6) numerically for various combinations of $\eta_{j}$, *P*_*f*_, and *P*_*d*_. The EC is then trained on this dataset to predict sensing samples, eliminating the need to solve the non-convex equation in real time.

These estimated samples will lead to minimum error probabilities and higher-throughput interpretation in the FC. A feature vector of the dataset is represented as

$$x=\left[\begin{array}{cccc} \eta_{j} & P_{f} & k & P_{d} \end{array}\right].$$ (7)

This allows the EC to be trained with the $\left(\eta_{j},\;P_{f},\;P_{d}\right)$ input and *k*_*j*_ output labels.

### 3.2 Classification with the AdaBoost

The EC creates a more reliable classifier by ensembling the contents of multiple weak classifiers and mixing their results with more promising prediction results. The ensembling method allows weak classifiers to update and eliminate retraining requirements. As individual classifiers result in prejudiced predictions, ensembling the categories of different classifiers is a more admissible choice. The execution of the EC test is compared with the DT, GNB, NN, KNN, RUSBoost, and XGBoost algorithms to select optimal sensing as required.

The users reporting energies form a history matrix at the FC as

$$T={\left[\begin{array}{cccc} t_{1} & t_{2} & \ldots & t_{n} \end{array}\right]}^{T},i\in \left\{1,2,...,n\right\},$$ (8)

where *t*_1_,*t*_2_ and *t*_*n*_ denote the *n* feature vectors that have the detection, false alarm and SNR requirements as the input and sensing samples as the response or output. The AdaBoost constructs stronger classifiers with an ensemble of weak classifiers. The training set *T* is an×(m+1) matrix in the space $T\in {ℜ}^{n\times (m+1)}$ as

$$T=\left[x_{i}|y_{i}\right],i\in \left\{1,...,n\right\},$$ (9)

where *x*_*i*_ denotes the input feature vectors to be classified with the ensembling method. The input training data to the EC in matrix form *T* has *X* and *Y* submatrices. Here, *X* is the *n* feature matrix, and *Y* is the *n* × 1 output label matrix that constructs the sensing samples.

Thus, a reduction in the sensing duration $\tau_{j}$ leads to a higher throughput since more time is allocated for data transmission. As $\tau_{j}=\frac{k_{j}}{f_{s}}$ depends on the sensing samples *k*_*j*_ (with *f*_*s*_ fixed), reducing *k*_*j*_ directly shortens $\tau_{j}$ and improves throughput. However, this reduction comes at a cost: fewer sensing samples degrade detection reliability, increasing the probability of missed detections (*P*_*m*_) and false alarms (*P*_*f*_).

In conventional non-configurable CRNs, *k* is fixed without accounting for dynamic sensing conditions, often leading to excessive sensing (reducing throughput) or insufficient sensing (compromising detection). In contrast, the proposed reconfigurable network optimizes *k*_*j*_ adaptively using EC, balancing throughput and detection accuracy based on real-time SNR, *P*_*f*_ requirements, and *P*_*d*_ constraints. For example, under favorable SNR conditions, *k*_*j*_ can be safely reduced to boost throughput without violating the thresholds of *P*_*d*_, whereas in low-SNR regimes, *k*_*j*_ is increased to maintain reliable detection.

The energy consumed during sensing, given by

$$s(i)=\sum_{j=1}^{m}\left(E_{j}(i)\times \tau_{j}\right),$$ (10)

further underscores this trade-off: while lower *k*_*j*_ reduces *s*(*i*), it risks higher sensing errors. Thus, the proposed scheme dynamically optimizes *k*_*j*_ to ensure that throughput gains do not come at the expense of unacceptable sensing performance, adhering to predefined *P*_*f*_ and *P*_*d*_ targets.

### 3.3 Data denoising through autoencoder

**Fig 5:**
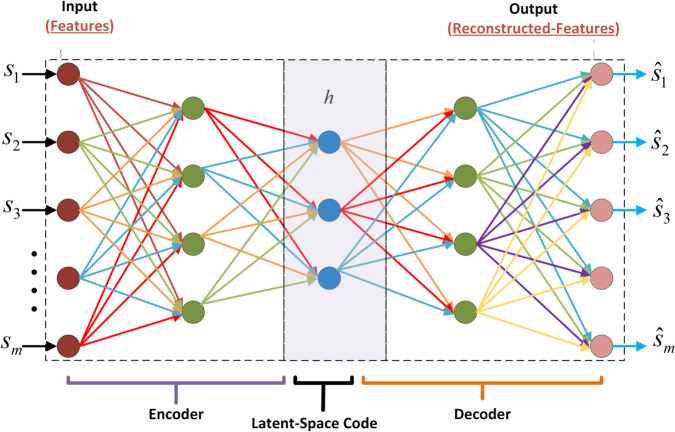
DAE.

A DAE is used that reduces the dimensionality of the reported sensing data and reconstructs the data from anomalies due to noisy channel behavior and false reporting by NFS, YFS, OFS and YNFS. This work examined DAE as a variant of the simple autoencoder for better results. However, DAE offers more features than the simple autoencoder in reconstructing the sensing reports from the attacked or corrupted sensing information. Fig 5 shows the architecture of the DAE, which consists of three main components: The encoder, decoder, and compressed latent space. The encoder section transforms the corrupted and attacked input *s* into the hidden and latent space representation *h* using the non-linear transformation.

$$h=f\left(W_{1}s+b\right).$$ (11)

This *f* is a nonlinear activation function, such as a logistic sigmoid (logsig) and rectified linear unit (ReLU) function. $W_{1}\in IR^{n\times m}$ is the weight matrix and $b\in IR^{n}$ is optimized in an encoder with *n* nodes in latent space. Then, the decoder evolves the latent space into a reconstructed vector, $\hat{s}$, at the output layer, employing nonlinear adaptation as follows:

$$\hat{s}=g\left(W_{2}s+c\right),$$ (12)

where *g* denotes the pure linear activation function (purelin). To improve learning efficiency, $W_{1}={W_{2}}^{T}$. The reconstruction error is calculated for a given input training set ${\left\{s_{i}\right\}}_{i=1}^{n}$ as $\sum_{i=1}^{n}{\left\|s_{i}-{\hat{s}}_{i}\right\|}^{2}$. The DAE training objective is to find the optimal parameters $\psi =\left\{W_{1},\;\;b,\;\;c\right\}$ that minimize the reconstruction error, as shown below:

$$\underset{\psi }{\min}\;\sum_{i=1}^{n}{\left\|s_{i}-{\hat{s}}_{i}\right\|}^{2}.$$ (13)

It is evident from (13) that the reconstruction error is the dissimilarity between the actual sensing data and the reconstructed output.

### 3.4 Channel state final decision

**Fig 6:**
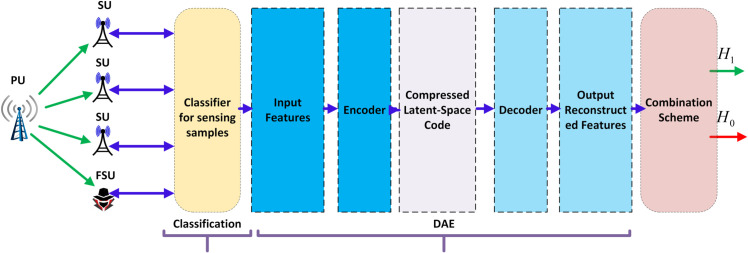
Sample estimation and report cleaning using a DAE.

Here, the system makes its final decision on channel availability using various combination schemes as in Fig 6. The identical gain combination (IGS) allocates equal weightage to the individual user reports and deals with the users similarly in constructing its decision. Similarly, the highest-gain scheme (HGS) assigns high weight to the user with high channel gain. The cumulative decision of the system using the IGS combination is as follows:

$$C_{IGS}(i)=\left\{\begin{array}{c} H_{1}:\;\;\;\;\;\;\;\;\;\;\frac{1}{m}\sum_{j=1}^{m}s_{j}(i)\geq \gamma \\ H_{0}:\;\;\;\;\;\;\;\;\;\;otherwise \end{array}\right\}.$$ (14)

The detection and false alarm probabilities $P_{d\;\;\;\_\;\;\text{ IGS}}$ and $P_{f\;\;\;\_\;\;\text{ IGS}}$ of the IGS according to the cumulative decision *C*_*IGS*_(*i*) are given by

$$P_{d\;\;\;\_\;\;\text{ IGS}}=\mathrm{Pr}\left\{\frac{1}{m}\sum_{j=1}^{m}s_{j}(i)\geq \gamma |H_{1}\right\},\;P_{f\;\;\;\_\;\;\text{ IGS}}=\mathrm{Pr}\left\{\frac{1}{m}\sum_{j=1}^{m}s_{j}(i)\geq \gamma |H_{0}\right\}.$$ (15)

The HGS combination multiplies each user-received report by its branch gain. Therefore, a higher weight is assigned to the user sensing with a high SNR regime and a lower weight to the user sensing with a low SNR regime. The cumulative decision *C*_*HGS*_(*i*) at the FC using the HGS technique is written as

$$C_{HGS}(i)=\left\{\begin{array}{c} H_{1}:\;\;\sum_{j=1}^{m}\;\left(w_{j}\times s_{j}(i)\right)\;\geq \gamma \\ H_{0}:\;\;otherwise \end{array}\right\},$$ (16)

where $w_{j}=\frac{\eta (j)}{\sum_{j=1}^{m}\eta (j)}$. The cooperative detection and false alarm probabilities of the HGS scheme are measured based on the individual sensing reports as

$$P_{d\;\;\;\_\;\;\text{ HGS}}=\left\{\frac{1}{m}\sum_{j=1}^{m}\left(w_{j}\times s_{j}(i)\right)\geq \gamma |H_{1}\right\},\;P_{f\;\;\;\_\;\;\text{ HGS}}=\left\{\frac{1}{m}\sum_{j=1}^{m}\left(w_{j}\times s_{j}(i)\right)\geq \gamma |H_{0}\right\}.$$ (17)

Unlike the IGS and HGS combinations, this scheme forms its cumulative determination using IGS through reconstructed data ${\hat{s}}_{i}$ at the output. The cumulative decision of the ECDAE at the FC is as follows:

$$C_{ECDAE}(i)=\left\{\begin{array}{c} H_{1}:\frac{1}{m}\sum_{j=1}^{m}{\hat{s}}_{j}(i)\geq \gamma \\ H_{0}:otherwise \end{array}\right\},$$ (18)

where *C*_*ECDAE*_ is the cumulative response of the ECDAE. The detection and false alarm probabilities of the ECDAE are expressed as

$$P_{d\;\_\text{ ECDAE}}=\mathrm{Pr}\left\{G_{ECDAE}(i)=1|H_{1}\right\},\;P_{f\;\_\text{ ECDAE}}=\mathrm{Pr}\left\{G_{ECDAE}(i)=1|H_{0}\right\}.$$ (19)

## 4 Simulation results and discussions

In this section, we first present the performance evaluation of the distinct ML classifiers to estimate the sensing samples for the users, taking into account the target demands of the system. The reconfigured sensing time is then employed in spectrum sensing for cooperative users. The users then perform sensing based on the reconfigured sensing time for the target detection, false alarm, and SNRs expected from the cooperative SUs. Finally, the DAE scheme cleans the sensing contents of the FSU disturbance. The total number of cooperative users making the dataset is set to 10, as in Table 3. The SNRs in the dataset formation range from -15 dB to 4 dB.

**Table 3:** Simulation parameters.

Parameter	Value
Total sensing iterations	1000
Total sensing users	10
SNRs	-15 dB to 4 dB
False alarm levels	0.01 to 0.11
Target detection probability levels	0.01 to 0.99
Dataset features for ML	100,000
Dataset features for DAE	20,000
Total frame period	100 ms
Bandwidth	40 KHz

All comparative models, including PSO [31], DE-ML [32], and CNN [25], were implemented and their parameters optimized following the best practices outlined in their respective source publications to ensure a fair comparison.

The performance of the proposed ECDAE framework is evaluated through comprehensive simulations. The results are structured to first detail the dataset creation process, followed by a comparative analysis of classifiers for sensing-time optimization, an evaluation of the DAE’s denoising efficacy, and finally, the overall sensing performance under various attack scenarios.

### 4.1 Dataset creation

To train and evaluate the proposed framework, synthetic datasets were generated for classifier selection and DAE to emulate realistic CRN conditions under both normal operation and false attacks. The dataset for classifier selection is generated by solving (6) numerically for various combinations of $\eta_{j}$, *P*_*f*_, and *P*_*d*_. This allows us to create a lookup table that the EC can use to predict sensing samples for target detection probability, false alarm, and SNR conditions. The dataset for the ML classifiers to predict sensing samples consists of ten levels of false alarm probability ranging from 0.01 to 0.11 (step size 0.01) with a target detection probability ranging from 0.01 to 0.99 (step size 0.1). In the formation of the dataset, the SNRs change from –15 dB to 4 dB (step size 1 dB) in the presence of ten sensing users. The corresponding number of samples is determined by a combination of these values. Hence, the sensing samples are collected for 50 sensing iterations to obtain 100,000 examples in the dataset. The classifiers are trained through the dataset with the detection probability, the false alarm probability, and the SNRs as input and the number of samples as output features. The signal bandwidth and sampling frequency values are selected at 40 kHz with a frame duration of 100 ms.

**Table 4:** Dataset schema for classifier training.

Feature	Description	Range/Values
$\eta_{j}$	Signal-to-Noise Ratio of the *j*^*th*^ user	-15 dB to 4 dB (1 dB steps)
*P*_*f*_	Target false alarm probability	0.01 to 0.11 (0.01 steps)
*P*_*d*_	Target detection probability	0.01 to 0.99 (0.1 steps)
*k*	**Output:** Optimal number of sensing samples	Predicted by the EC

The dataset comprises 100,000 samples generated by evaluating all combinations of the input parameters $\eta_{j}$, *P*_*f*_, and *P*_*d*_.

Similarly, the dataset for DAE consists of 20,000 feature vectors, each containing energy readings from 10 cooperative SUs (columns 1-10) and a binary PU state label (column 11). PU states (*H*_0_: absent, *H*_1_: present) were generated with perfect ground-truth knowledge using a balanced distribution ($P(H_{1})=P(H_{0})=0.5$). The energy values were simulated according to Gaussian distributions: $𝒩(\mu_{0}=k,\sigma_{0}^{2}=2k)$ under *H*_0_ and $𝒩(\mu_{1}=k(\eta_{j}+1),\sigma_{1}^{2}=2k(\eta_{j}+1))$ under *H*_1_, with SNRs $\eta_{j}$ varying from -15dB to 4dB between users to ensure diversity.

The dataset incorporates two attack configurations: (1) fixed position attacks where specific columns (e.g., 5-7) consistently contained FSUs and (2) random position attacks where FSUs were dynamically assigned per sensing interval. Three attack types were modeled: NFS users consistently report *H*_0_ energies (𝒩(k,2k)), YFS users always transmit *H*_1_ energies ($𝒩(k(\eta_{j}+1),2k(\eta_{j}+1))$), and YNFS attackers randomly switch between both strategies. The input data was split into 80% training sets and 20% test sets. To improve DAE robustness, Gaussian noise (noise level = 0.15) was added to the training data. Table 5 summarizes the complete structure of the DAE dataset. The complete dataset used for DAE training is available in S4 File.

**Table 5:** Dataset schema for DAE.

Col	Desc.	Type	Normal (*H*_0_)	Normal (*H*_1_)	FSU (*H*_0_)	FSU (*H*_1_)
1-10	Energies	Float	*N*(*k*,2*k*)	N(kζ,2kζ)	NFS: *N*(*k*,2*k*); YFS: N(kζ,2kζ); OFS: N(kζ,2kζ); YNFS: Mixed	NFS: *N*(*k*,2*k*); YFS: N(kζ,2kζ); OFS: *N*(*k*,2*k*); YNFS: Mixed
11	PU State	Binary	0	1	0	1

$N(\mu ,\sigma^{2})$: Gaussian distribution; ζ=(η+1); η: SNR; *k*: scaling factor. Each row corresponds to one sensing interval.

### 4.2 Classifier selection

**Fig 7:**
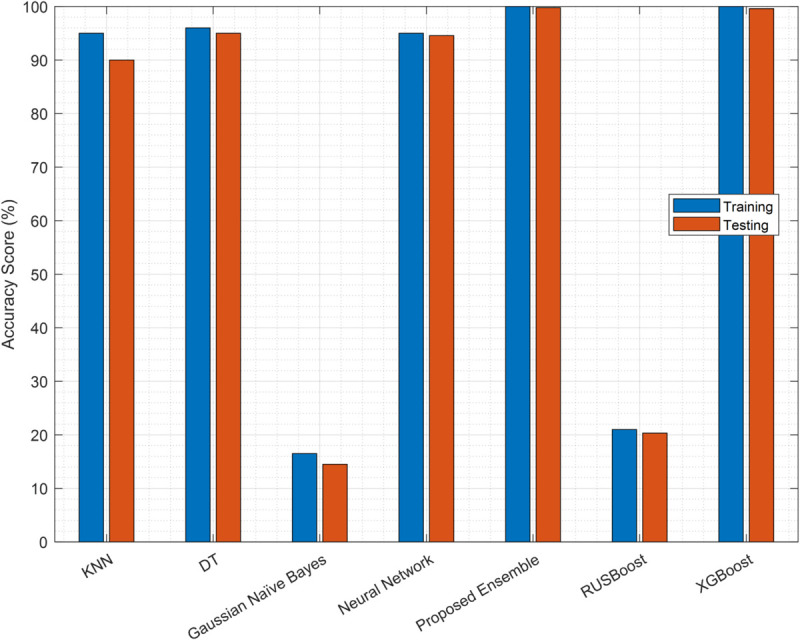
Classifier accuracy performance.

We first evaluate the performance of multiple candidate classifiers to identify the most accurate and reliable model. In this section, multiple classifiers are evaluated to verify their ability to classify sensing samples. The accuracy results in Fig 7 demonstrate the superiority of the EC, achieving an accuracy of 100% training and an accuracy of 99.78% in the testing, closely followed by XGBoost (100% training, 99.6% testing). Among traditional classifiers, DT performs well (96% training, 95% testing), while NN and KNN also show competitive results (95% training, 94.56% testing and 95% training, 90% testing, respectively). In contrast, GNB and RUSBoost exhibit significantly poorer performance (16.5%/14.5% and 21%/20.33%, respectively), highlighting their unsuitability for this task.

**Fig 8:**
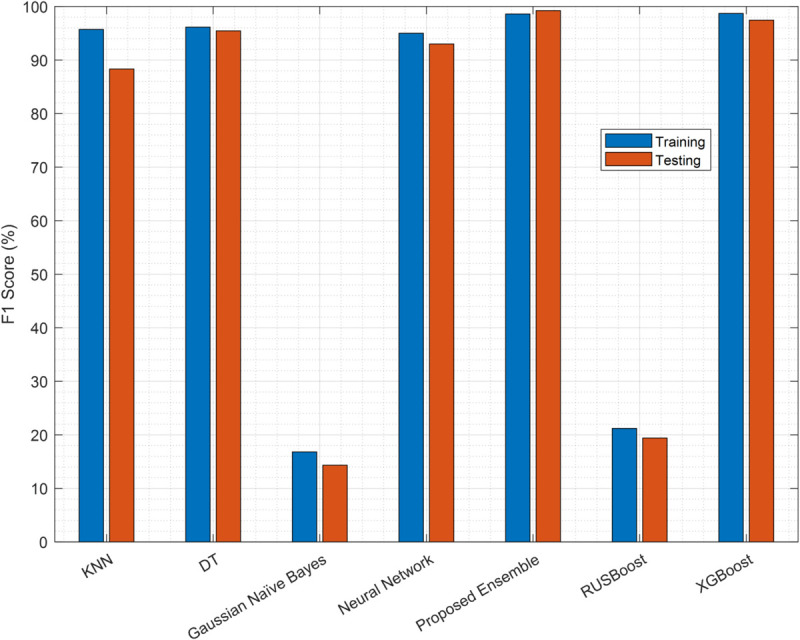
Classifier F1-score performance.

The results of the F1 score in Fig 8 further validate the dominance of the EC, with a training of 98.6% and 99.23% testing, outperforming XGBoost (98.7% training, 97.45% testing) and other baselines. DT (96.14% training, 95.45% testing) and NN (95% training, 93% testing) maintain robust performance, while KNN shows a notable drop in the F1 test score (88.32% vs. 95.71% training). GNB and RUSBoost again under-perform, reinforcing their limitations in this context.

**Fig 9:**
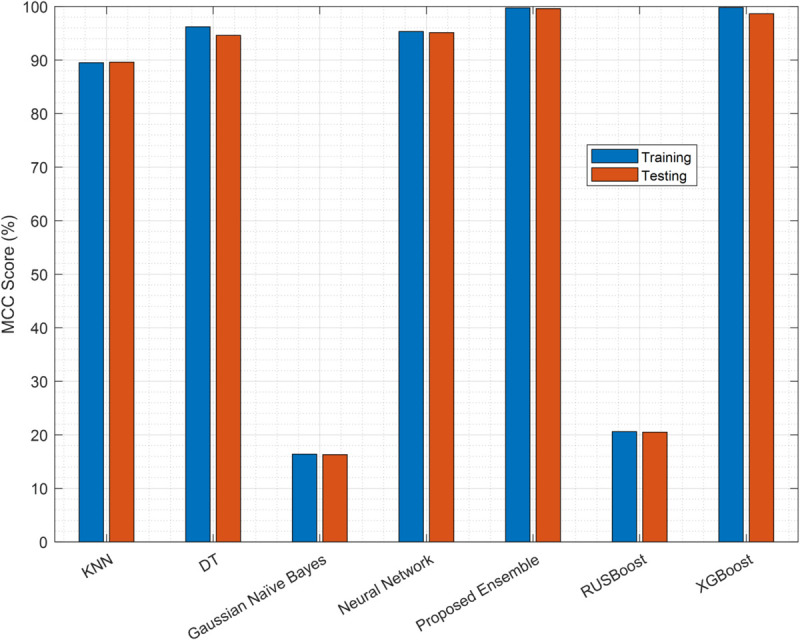
Classifier MCC performance.

The MCC results in Fig 9 assess the authenticity of the classifier, with the EC method achieving near perfect scores (99.74% training, 99.6% testing), marginally surpassed by XGBoost in training (99.8%) but superior in testing. DT and NN exhibit strong consistency (DT: 96.2%/94.61%; NN: 95.33%/95.12%), while KNN’s MCC aligns with its accuracy (89.5%). GNB and RUSBoost produce the lowest MCC values (16% and 20%), corroborating their unreliability.

The EC consistently outperforms others in all metrics (accuracy, F1 score, MCC), with XGBoost as a close competitor. DT and NN are viable alternatives, whereas GNB and RUSBoost are not. Although XGBoost demonstrates competitive performance (99.6% accuracy, 97.45% F1 score), our proposed EC (99.78% accuracy, 99.23% F1 score) is preferred for deployment in CRNs due to three key advantages: (1) lower computational complexity during both training and inference, making it suitable for resource-constrained edge devices; (2) simpler hyperparameter tuning compared to XGBoost’s extensive parameter space; and (3) inherently more interpretable decision-making through its linear combination of weak learners with explicit weights, facilitating better model transparency and debugging in practical CRN deployments.

The high accuracy of the EC reflects its success in learning the deterministic mapping from the input parameters to the optimal sensing samples k, a well-defined regression task for which near-perfect performance is achievable. Furthermore, the DAE’s performance was rigorously evaluated on a separate, unseen test set, and we employed L2 and sparsity regularization (as described in Sect 4.3) specifically to mitigate overfitting.

Having identified the EC as the optimal model for predicting samples, these samples are now used by the SUs to perform sensing. The resulting reports, which may be corrupted by noise and FSUs, form the input for the next stage of our denoising framework. The completenumerical results for classifier performance (Figs 7–9) are available in S2 File.

### 4.3 DAE architecture and performance

**Fig 10:**
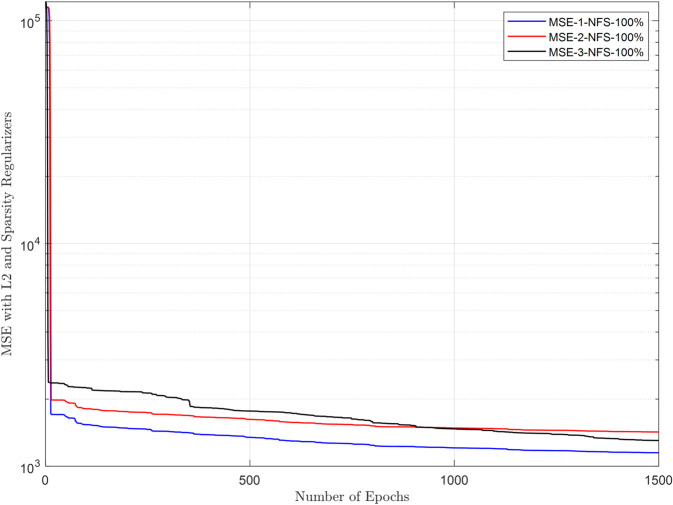
MSE vs. epoch in the presence of NFS.

**Fig 11:**
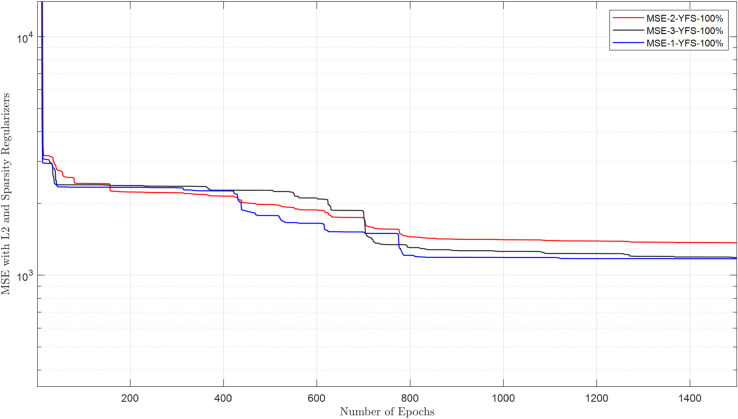
MSE vs. epoch in the presence of YFS.

**Fig 12:**
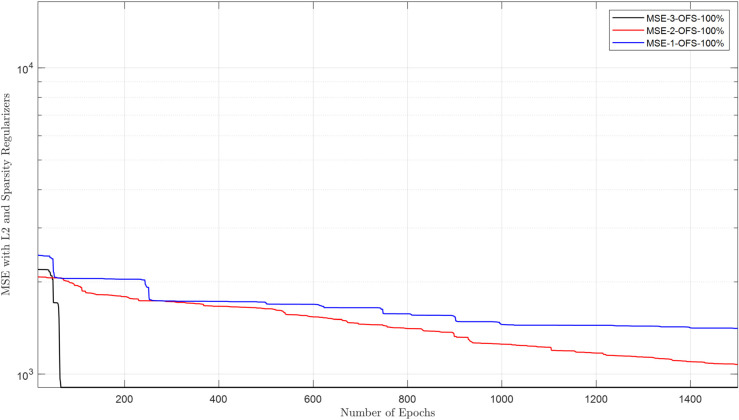
MSE vs. epoch in the presence of OFS.

**Fig 13:**
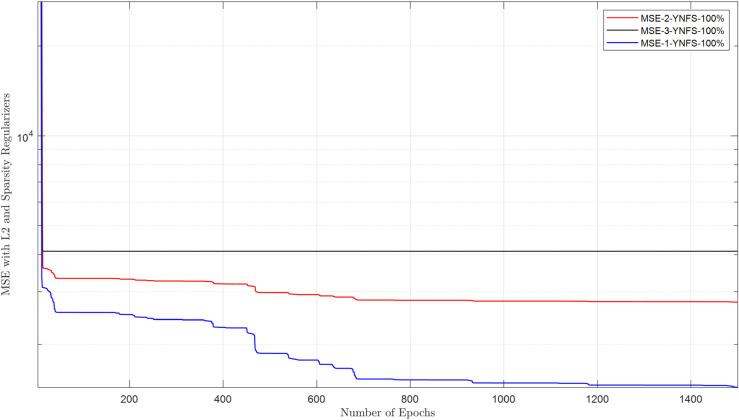
MSE vs. epoch in the presence of YNFS.

The raw energy reports collected in the FC are purified using a DAE. This section evaluates the DAE architecture and its ability to reconstruct clean data from inputs corrupted by channel noise and false sensing attacks. The cooperative user uses the estimated sensing samples through the EC for sensing. The DAE architecture employs a 2-neuron hidden layer, optimized for generalization, with logsig (encoder) and purelin (decoder) transfer functions to handle bounded energy values and linear reconstruction, respectively. Training spans 1500 epochs using a combined MSE-sparse loss function (msesparse), strengthened by regularization of L2 (0.01) and sparsity constraints (proportion = 0.1, regularization = 1) to prevent overfitting. Reconstruction performance is evaluated under adversarial conditions, where NFS users (always reporting low energy) and YFS users (always reporting high energy) attack at rates of 10%–100%. Figs 10–13 demonstrate that the DAE achieves stable convergence, with loss values decreasing monotonically as the epochs increase. Fig 10 shows MSE results in the presence of 1, 2, and 3 NFS users with 100% attacks that always report false low energy reports in both hypotheses. The results in the figure show a decrease in MSE as the number of epochs increases from 1 to 1500. Similarly, the MSE results for DAE are collected in the presence of YFS users in Fig 11, which shows a reduction in the loss function as the number of epochs increases and less impact when the YFS increases from 1 to 3. The DAE MSE results are collected in Fig 12 in the presence of OFS users that report opposite information on channel availability compared to actual states. In the final check to confirm the denoising effect of DAE, a probabilistic attack from the YNFS users is tested in Fig 13. In this kind of attack, the attacker switched between the NFS and the YFS attacker probabilistically. The figure shows a slight increase in the loss results when the number of FSU attackers increases from 1 to 3 attacking at a rate of 100%. The results of the loss function show that the system can reconstruct the data with a minimum error in the presence of NFS, YFS, OFS, and YNFS users.

The stable convergence and low reconstruction error demonstrate the proficiency of the DAE in cleaning the sensing data. This cleaned data is subsequently used for the final decision fusion at the FC.

### 4.4 Ablation study: Component-wise performance

To quantitatively isolate and evaluate the contribution of each component of the proposed ECDAE framework, an ablation study was conducted. This analysis compares the performance of the EC-only model, the DAE-only model, the full hybrid ECDAE model, and a baseline PCA denoising method. The goal is to demonstrate that the synergy between dynamic sensing-time optimization and data denoising is crucial to achieving better performance and that neither component alone is sufficient.

The results, summarized in Table 6, were obtained under a challenging scenario with 3 FSUs attacking at a 100% rate and an SNR of -5 dB. The key metrics are the final decision error probability (*P*_*e*_) for each type of attack and the computational latency.

**Table 6:** Performance comparison of individual components versus the full ECDAE framework.

Metric	PCA-Only	DAE-Only	EC-Only	ECDAE
*P*_*e*_ (NFS)	0.0051	0.0012	0.0040	0.0002
*P*_*e*_ (YFS)	0.0152	0.0008	0.0035	0.0004
*P*_*e*_ (OFS)	0.0083	0.0021	0.0048	0.0005
*P*_*e*_ (YNFS)	0.0062	0.0015	0.0042	0.0003
Latency (ms)	0.3	0.7	1.1	**1.4**

Results are for a scenario with 3 FSUs at 100% attack rate and SNR = -5 dB. Error probability (*P*_*e*_) is defined in Eq (20). Latency represents the average added processing time per sensing interval. The PCA-only scheme applies Principal Component Analysis for dimensionality reduction and reconstruction as a denoising baseline.

**Fig 14:**
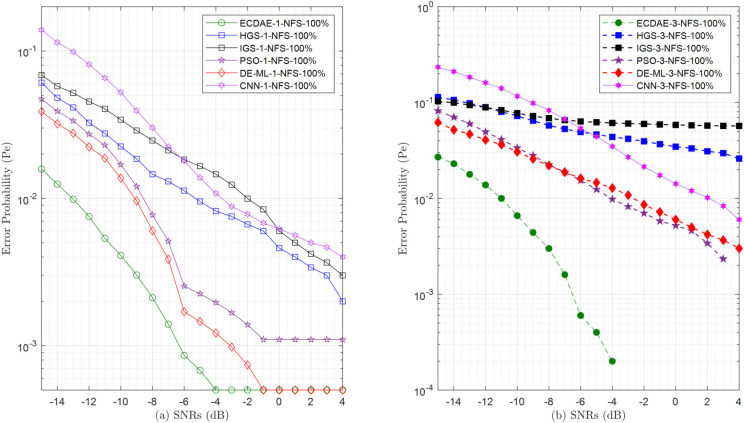
Error probability (Pe) vs. SNRs (dB) for (a) one NFS attack (b) three NFS attack.

**Fig 15:**
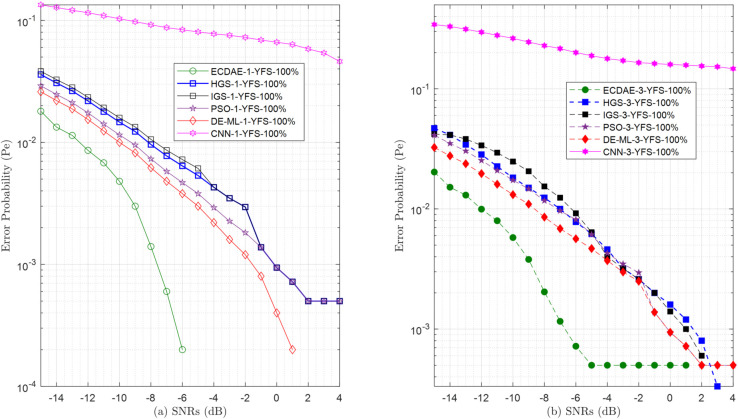
Error probability (Pe) vs. SNRs (dB) for (a) one YFS attack (b) three YFS attack.

**Fig 16:**
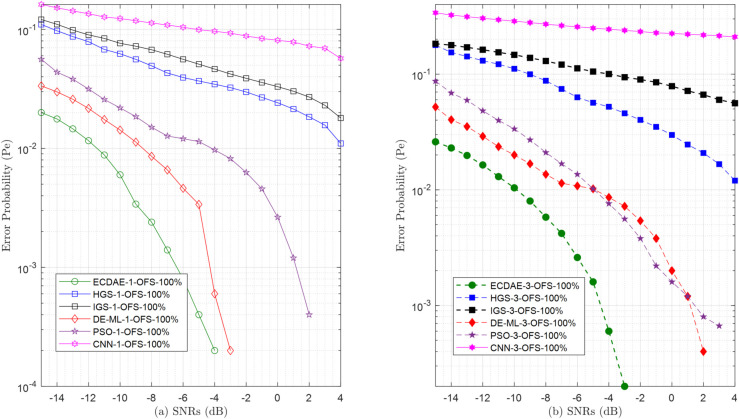
Error probability (Pe) vs. SNRs (dB) for (a) one OFS (b) three OFS attack.

**Fig 17:**
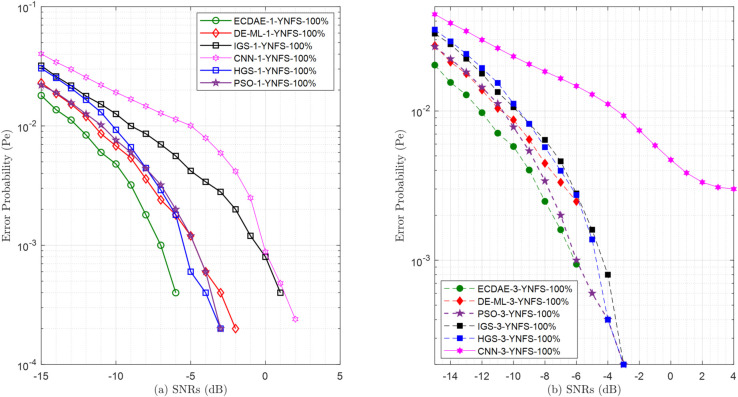
Error probability (Pe) vs. SNRs (dB) for (a) one YNFS attack (b) three YNFS attack.

The results lead to several critical insights:

- EC-Only Limitations: The EC-Only scheme, which only adapts the sensing time but does not apply denoising, struggles against FSUs. Although it improves reliability, it has no mechanism to cleanse the corrupted data reports, resulting in the highest error probabilities across all types of attack (e.g., *P*_*e*_ = 0.004 for NFS).
- PCA-Only Baseline: The PCA-only scheme, a linear dimensionality reduction technique, serves as our denoising baseline. It reduces error compared to the raw data (e.g., see IGS/HGS results in Figs 14–17) but is significantly outperformed by all other learning-based components. Its linear transformation is insufficient to capture the complex, non-linear distortions introduced by both the channel and malicious FSU attacks, resulting in the highest error probabilities among the compared components (e.g., *P*_*e*_ = 0.0051 for NFS).
- DAE-Only Limitations: The DAE-Only scheme, which denoises the data but uses a fixed, non-optimized sensing time, performs better than EC-Only. It effectively mitigates the impact of FSUs, as seen in the lower error rates (e.g., *P*_*e*_ = 0.0012 for NFS). However, its performance is limited by the suboptimal quality of the input data; without an optimized sensing time, the energy reports consume more sensing time, making the DAE’s reconstruction task difficult.
- DAE-Only vs. PCA: The DAE-Only scheme, which employs a non-linear denoising autoencoder, significantly outperforms the linear PCA baseline (e.g., a 76% reduction in error for NFS attacks). This performance gap highlights the advantage of the DAE’s non-linear activation functions and learned representations in effectively isolating false data patterns and noise.
- ECDAE performance: The full ECDAE framework delivers the best performance, reducing the error rate by 5 to 10 more compared to the best standalone component. This shows a powerful sequential integration: the EC front-end provides optimally clean data by configuring the sensing duration, which in turn makes the DAE back-end significantly more effective at purification and reconstruction. The resulting error probabilities are near zero across all attack types.
- Latency Overhead: The added computational latency of the full ECDAE pipeline (1.4 ms) is the sum of its parts but remains well within the budget of a typical 100 ms sensing frame (see Table 3). This minimal overhead is a worthwhile trade-off for the improvement in sensing reliability and security.

The ablation study confirms that both EC and DAE components are essential, with their sequential integration creating a synergistic relationship rather than merely additive performance gains. This synergy—where the EC front-end provides optimally configured sensing data that enhances the DAE back-end’s purification effectiveness—enables robust cross-generalization across diverse attack scenarios, a key advantage over standalone components.

### 4.5 Final sensing performance

This section presents the overall *P*_*e*_ after processing the cleansed data using the soft combination rule, comparing our method against the state-of-the-art techniques for all types of attack. Performance is evaluated using the total probability of error (*P*_*e*_), which provides a balanced measure of the overall decision of the FC accuracy by combining the probabilities of false alarms and missed detections. For a given combination scheme, *P*_*e*_ is defined as:

$$P_{e}=\mathrm{Pr}(H_{0})\cdot P_{f}+\mathrm{Pr}(H_{1})\cdot P_{m}$$ (20)

where $\mathrm{Pr}(H_{0})$ and $\mathrm{Pr}(H_{1})$ are the prior probabilities of the PU being absent or present, respectively (assumed to be $\mathrm{Pr}(H_{0})=\mathrm{Pr}(H_{1})=0.5$ in our simulations), *P*_*f*_ is the false alarm probability from Eqs (15), (17), or (19), and *P*_*m*_ = 1 − *P*_*d*_ is the missed detection probability, with *P*_*d*_ being the detection probability. This metric *P*_*e*_ is plotted against the SNR in Figs 14–17 for all the evaluated schemes. ECDAE consistently demonstrates superior robustness across all scenarios, achieving substantially higher error probability reduction than competing methods, particularly at improved SNR conditions. The subsequent analysis quantifies these performance gains.

In the NFS attacks in Fig 14, the proposed ECDAE scheme demonstrates unparalleled robustness against varying numbers of FSUs. With three attackers (ECDAE-3-NFS), it achieves 0% error at SNR ≥−3 dB, while its closest competitors, PSO-3-NFS [31] and DE-ML-3-NFS [32], plateau at 0.003 and 0.0023, respectively. The CNN-3-NFS [19] approach, although better than traditional schemes, remains inadequate with three attackers, stagnating at 0.006 (6× higher than ECDAE) even at SNR  = 4 dB. In stark contrast, non-learning schemes fail catastrophically under coordinated attacks: IGS-3-NFS maintains a 0.057 error in the SNR  = 4 dB, which is worse than ECDAE 0%, while HGS-3-NFS marginally improves to 0.026.

Critically, the superiority of ECDAE holds even with fewer attackers: for a single MU (ECDAE-1-NFS), the error drops to 0.0005 at SNR  = 4 dB, outperforming HGS-1-NFS (0.002) and IGS-1-NFS (0.003). These results underscore ECDAE scalability, maintaining dominance under both low (1 attacker) Fig 14(a) and high (3 attackers) Fig 14(b) adversarial loads, while traditional schemes degrade catastrophically with increasing attackers.

For YFS attacks in Fig 15, where FSUs consistently report high energy signals regardless of actual spectrum conditions, the proposed ECDAE demonstrates definitive enhancement. With one attacker (ECDAE-1-YFS) Fig 15(a), perfect detection (0% error) is achieved at SNR ≥−5 dB, whereas DE-ML-1-YFS requires SNR ≥−1 dB to achieve zero error. At SNR  = −10 dB, ECDAE-1-YFS exhibits an error of 0.0048, compared to 0.0137 for DE-ML-1-YFS and 0.0291 for PSO-1-YFS.

In coordinated attacks with three FSUs Fig 15(b), conventional learning approaches fail catastrophically, sustaining 0.147 error even at favorable SNR (4 dB). Traditional schemes show fundamental limitations: HGS-1-YFS achieves zero error only when SNR is ≥2 dB, while IGS-3-YFS cannot suppress errors below 0.0159 at SNR  = −10 dB, more than three times higher than ECDAE-1-YFS’s 0.0048 under identical conditions.

In the OFS attack scenarios in Fig 16, ECDAE maintains its lead, reaching a 0% error at SNR ≥ –4 dB with single attacker Fig 16(a), 33% more SNR efficient than DE-ML-1-OFS, which achieves this only at SNR ≥ –3 dB. For three attackers Fig 16(b), ECDAE-3-OFS achieves a 0% error at SNR ≥ –3 dB, while DE-ML-3-OFS lags at SNR ≥ –1 dB. PSO-3-OFS shows moderate resilience but still exhibits a 0.0007 error in the SNR equal to 3 dB, which is worse than ECDAE 0%. The CNN-3-OFS scheme performs poorly, with an error that lingers at 0.21 (higher than ECDAE) at SNR equal to 4 dB. Meanwhile, traditional IGS and HGS schemes collapse under pressure: IGS-3-OFS error remains 0.056 higher than ECDAE at SNR = 4 dB, and HGS-3-OFS improves only to 0.012. Even with one attacker, HGS-1-OFS (0.011) and IGS-1-OFS (0.018) are worse, respectively, than ECDAE-1-OFS (0.0005). This confirms ECDAE’s unmatched capability to mitigate opportunistic attacks, where both learning and non-learning alternatives fall short.

For the complex YNFS attacks in Fig 17, ECDAE maintains its superiority, achieving 0% error at SNR ≥−5 dB with one attacker Fig 17(a). This is 400% more SNR efficient than DE-ML-1-YNFS, which reaches this at SNR ≥−1 dB. At SNR  = −10 dB, ECDAE-1-YNFS (0.0048) outperforms PSO-1-YNFS (0.0076) by 37% and CNN-1-YNFS (0.0191) by 298%. With three attackers Fig 17(b), ECDAE-3-YNFS achieves a 0% error at SNR ≥−1 dB, while PSO-3-YNFS requires SNR ≥3 dB. CNN-3-YNFS fails entirely, with the error stuck at 0.003 (higher than ECDAE) at SNR  = 4 dB. The traditional schemes IGS and HGS show partial improvements, but remain non-competitive: IGS-3-YNFS achieves an error of 0% at SNR ≥3 dB (400% behind ECDAE), while HGS-3-YNFS achieves this at SNR ≥−3 dB (200% behind). At SNR  = −10 dB, ECDAE-1-YNFS 0.0048 is 140% and 158% better than HGS-1-YNFS (0.0115) and IGS-1-YNFS (0.0124), respectively. These results solidify ECDAE’s position as the only scheme capable of neutralizing hybrid threats with near-perfect accuracy. The complete error probability results for all scenarios (Figs 14–17) are provided in S3 File.

### 4.6 Computational complexity and comparative analysis

A critical aspect of deploying any ML-based solution in resource-constrained CRN devices is its computational overhead. This section analyzes the complexity-robustness trade-off of the proposed ECDAE framework and provides a quantitative comparison with other state-of-the-art methods, including those based on DAE and deep learning.

The inference complexity of ECDAE is determined by its two core components. The EC complexity is O(30·d·m), where 30 is the number of weak learners (decision trees) in the AdaBoost ensemble, *d* is the average depth of a tree (approximately log(n) for *n* training samples), and *m* is the number of users. The DAE involves a forward pass through an encoder and decoder. For our architecture with a 10-dimensional input, a 2-neuron hidden layer, and a 10-dimensional output, the complexity is *O*(*m*), specifically involving two matrix-vector multiplications. The total complexity is additive: $O(m{)}_{DAE}$  +  $O(30\cdot d\cdot m{)}_{EC}$.

We benchmarked the average processing latency per sensing interval for our Python/NumPy implementation on a standard CPU. The results, alongside key comparative metrics, are summarized in Table 7.

**Table 7:** Computational and robustness trade-offs of CSS approaches.

Method	Complexity (FLOPs)	Latency (ms)	Overfitting Risk	Attack Robustness
IGS/HGS	*O*(*m*)	0.5	None	Low (fails at >20% attacks)
PSO [31]	O(p·m·k)	4.5	None	Medium (fails at 50-70% attacks)
DE-ML [32]	O(m·k+p)	3.9	Low	Medium (fails at 70-80% attacks)
CNN [25]	O(L·f·m)	4.1	Moderate	Medium (fails at ~50% YFS)
Stacking Ensemble [13]	O(E·C(m))	~5.0	Moderate	Not Evaluated
ECDAE (Ours)	O(m)+O(T·d·m)	3.2	Low	High (100% attacks)

*m*: Number of users; *p*: Population size (PSO); *k*: Samples; *L*: CNN layers; *f*: CNN filters; *E*: Number of ensemble models; *C*(*m*): Cost per model; *T*: Number of trees; *d*: Tree depth (≈log(n)).

Latency measured for *m* = 10 on a standard CPU. Overfitting risk for ECDAE is mitigated via regularization in the DAE and ensemble diversity in the EC.

As evidenced in Table 7, the proposed ECDAE framework strikes a superior balance between computational cost and robustness:

- Vs. Traditional Schemes (IGS/HGS): While adding moderate latency (3.2 ms vs. 0.5 ms), ECDAE provides a monumental leap in robustness, tolerating 100% attack rates from diverse FSU types where traditional schemes fail catastrophically with just 20% malicious users.
- Vs. Optimization-based ML (PSO, DE-ML): ECDAE offers lower latency and significantly higher robustness. PSO and DE-ML lack a dedicated denoising component, making them vulnerable to higher attack rates (50-80%).
- Vs. Other DL/ML Models (CNN [25], Stacking Ensemble [13]): ECDAE is more efficient and robust. The CNN approach [25], while powerful for feature extraction, has higher complexity (O(L·f·m)) and was shown in Figs 14–17 to be far less robust to FSUs. The stacking ensemble method [13] fuses hard decisions from multiple complex models, likely leading to higher latency and, as our results demonstrate, does not address the soft-data denoising challenge solved by our DAE.

The 3.2 ms latency of ECDAE is well within the budget of a typical 100 ms sensing frame, confirming its practical deployability. This analysis justifies the slightly higher computational cost as a necessary and worthwhile trade-off for achieving near-perfect sensing reliability in adversarial environments, a feat unmatched by existing alternatives.

Furthermore, as established in Sect 4.2, the EC component offers superior interpretability compared to more complex alternatives, providing an additional practical advantage for system deployment and debugging.

## 5 Conclusions

Spectrum sensing is an essential step in the CR duty cycle that allows entry into opportunistic spectrum resources. A reliable sensing decision system is critical to underrate interference for the licensee. CSS is expected to achieve adequate sensing results in Rayleigh fading environments. Although more time spent in the sensing process increases the chances of the SUs catching the free spectrum accurately, this will also reduce the user’s data transmission time, which causes reduced throughput. Therefore, the slow convergence of the CSS is reduced when the SUs immediately report to the FC with their decision for guaranteed sensing. The estimated sensing samples in this paper with the ensemble classifier lead to a reconfigurable sensing time that increases the channel throughput and improves the sensing reliability. The assumed cooperative environment in this paper consists of both honest SU and FSU. The users follow estimated sensing samples of the ensemble classifier while reporting their energies to the FC. These reports consist of disturbances of the wireless channel and FSUs. The acquired messages are cleaned and reconstructed with a DAE before using the combination schemes. Accuracy, F1 score, and MCC comparison are discussed for various ML classifiers, followed by the DAE loss function to measure the effectiveness of DAE reconstruction.

While this study demonstrates the efficacy of the ECDAE framework using comprehensive synthetic data, future work will focus on validating its performance with real-world RF signal datasets and in hardware testbeds to further confirm its generalizability and practical deployment potential.

## Supporting information

S1 File Energy statistics of the reporting users. Sensing users energy reports.
(XLSX)

S2 File Classifiers performance for Figs 7–9. Results in numerical form.
(XLSX)

S3 File Error probability results for Figs 14–17. Error probability results in numerical form.
(XLSX)

S4 File Data for denoising autoencoder (DAE) training. Data used for training DAE.
(XLSX)
